# Breast cancer patients in Nigeria: Data exploration approach

**DOI:** 10.1016/j.dib.2017.08.038

**Published:** 2017-09-01

**Authors:** Pelumi E. Oguntunde, Adebowale O. Adejumo, Hilary I. Okagbue

**Affiliations:** a Department of Mathematics, Covenant University, Ota, Ogun State, Nigeria; b Department of Statistics, University of Ilorin, Ilorin, Kwara State, Nigeria

**Keywords:** Breast cancer, Logistic regression, Mortality, Oncology

## Abstract

Breast cancer is the type of cancer that develops from breast tissue; it is mostly common in women and it is one of the most studied diseases, largely because of its high mortality (second to lung cancer). However, it occurs in males also. This article presents a statistical study of the distribution of age, gender, length of stay, mode of diagnosis, status (dead or alive) after treatment and the location of breast cancer among 300 patients admitted in the University of Ilorin teaching hospital, Ilorin, Nigeria. The study covers a period of five (5) years; from 2011 to 2016 and logistic regression was used to perform the basic analysis in this study. It was discovered that the age of patients and the location of the breast cancer (right or left) contributes significantly to the survival of the patients. However, early detection and treatment of the disease is highly encouraged. This study also recommends that awareness should be taken to the grassroots and males should not be excluded from this discussion.

**Specifications Table**

**Table:** 

Subject area	Medicine
More specific subject area	Biostatistics, Oncology
Type of data	Table and text file
How data was acquired	Unprocessed secondary data
Data format	Raw, analyzed
Experimental factors	Records of Breast cancer patients obtained from University of Ilorin Teaching Hospital (UITH), Nigeria.
Experimental features	Computational Analysis: Histogram, Bar-chart, Contingency tables, Logistic regression analysis.
Data source location	University of Ilorin Teaching Hospital (UITH), Nigeria
Data accessibility	All the data are available in this data article as supplementary materials

**Value of the data**

• The data on breast cancer could be useful for government and health workers to make decisions that would reduce the risk of breast cancer among the populace.
• The data provides the analysis of the age, gender, location of the breast cancer, mode of diagnosis, length of stay (LOS), outcome of treatment of breast cancer patients for the population studied.
• The data can further be analyzed using other statistical tools like chi square test, multiple linear regression and Poisson regression analysis.
• The result from the analysis can be compared with other oncologic studies.
• The interpretation of the data could be helpful in educational studies, epidemiologic oncology, molecular pathologic epidemiology, and breast cancer awareness, screening and so on.
• The study can be replicated or extended to longitudinal studies.
• The article provides insight on the impact and consequence of age and location of breast cancer on the survivability of breast cancer patients.

## 1 Data

The data set used in this article was collected as a secondary data and it contains information on 300 breast cancer patients. The data set was obtained from the Cancer Registry Department under the Department of Admission and Discharge Unit, University of Ilorin Teaching Hospital (UITH) Ilorin, Nigeria. It involves information on 275 females and 25 males and it covers a period of five (5) years; from 2011 to 2016. The patients were all treated as in-patients and were later discharged, of these, 97 patients were discharged dead while 203 patients were discharged alive. The raw data is available and can be assessed as Supplementary data.

Descriptive analyses were performed and logistic regression analysis was also used to describe and analyze the data set.

The data is summarized under different classifications: gender (sex), location of the breast cancer, mode of diagnosis, survival after treatment, age and length of stay in the hospital during treatment.

### 1.1 Analysis of age of the patients

The frequency table showing the analysis of the age of all the 300 patients is shown in Table 1.

**Table 1:** Analysis of age.

**Statistics**		
Age		
		
N	Valid	300
	Missing	0
Mean		49.71
Median		50.00
Mode		60
Std. Deviation		13.884
Variance		192.768
Skewness		.572
Std. Error of Skewness		.141
Kurtosis		.479
Std. Error of Kurtosis		.281
Minimum		20
Maximum		96
		
Percentiles	25	40.00
	50	50.00
	75	60.00

In Table 1, it can be seen that the mean age of the patients is 49.71 years, the minimum and maximum ages are 20 years and 96 years respectively. The data set is slightly positively skewed with a coefficient of skewness of 0.572.

**Fig. 1:**
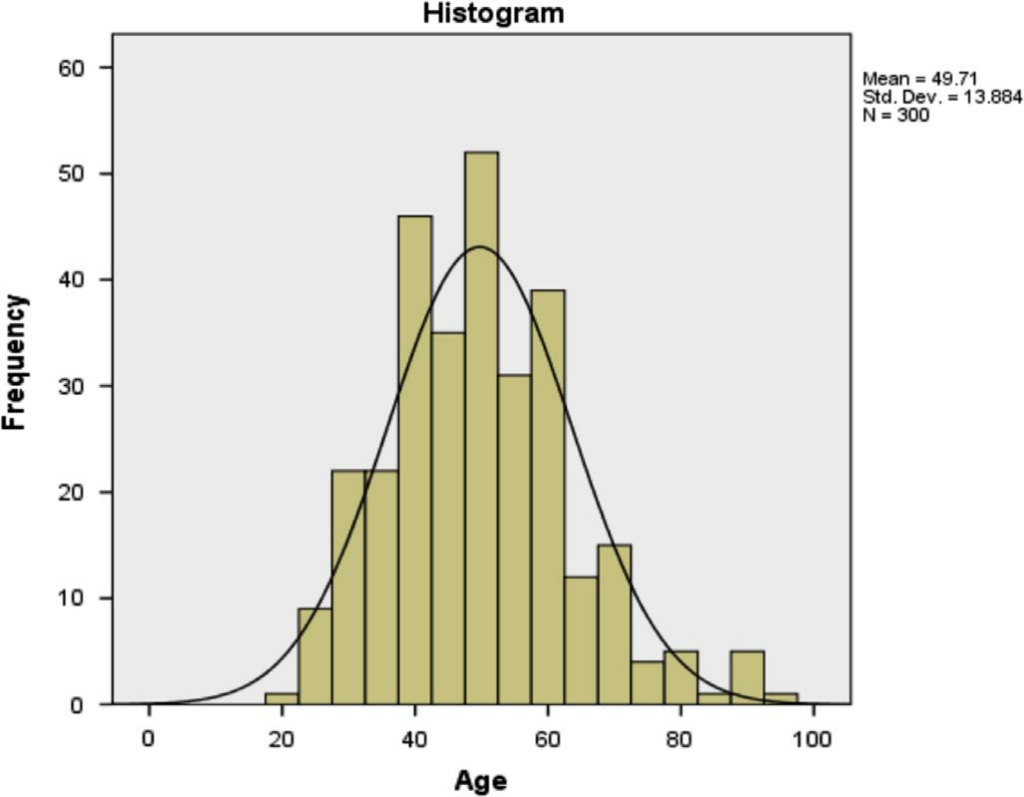
The distribution of age using histogram.

A diagrammatic representation of the age of the patients is as shown in Fig. 1.

The age of the patients were classified into three different groups (or classes) and the respective frequencies are as shown in Table 2.

**Table 2:** Classification of age of the patients.

**Agecode**					
		Frequency	Percent	Valid Percent	Cumulative Percent
Valid	<41years	88	29.3	29.3	29.3
	41–55years	115	38.3	38.3	67.7
	> 55years	97	32.3	32.3	100.0
	Total	300	100.0	100.0	

It can be seen from Table 2 that majority (115) of the patients are in the age group 41–55 years which accounts for 38.3% of the total population under study.

**Fig. 2:**
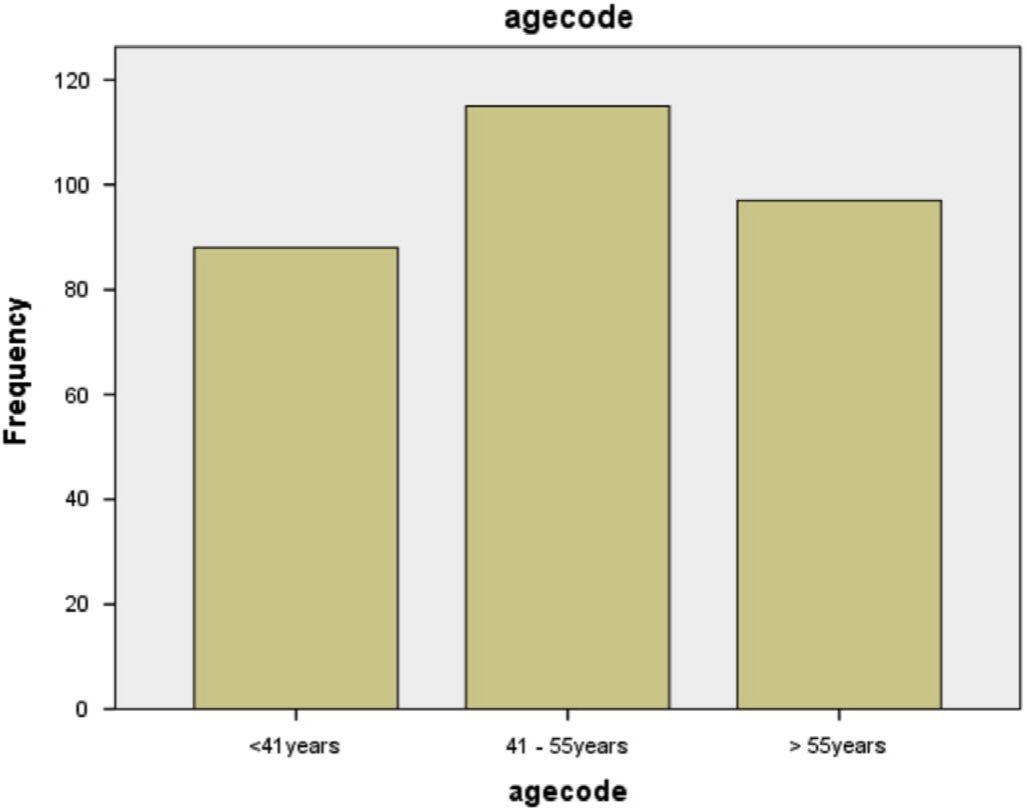
Bar chart showing the classification of age.

The diagrammatic representation of the information in Table 2 is as shown in Fig. 2.

### 1.2 Analysis on length of stay of the patients at the hospital

Information on the length of stay of the patients in the hospital before discharge is as shown in Table 3 and the respective frequencies are also displayed.

**Table 3:** Classification of length of stay.

**Loscode**					
		Frequency	Percent	Valid Percent	Cumulative Percent
Valid	< 11days	106	35.3	35.3	35.3
	11–21days	101	33.7	33.7	69.0
	> 21days	93	31.0	31.0	100.0
	Total	300	100.0	100.0	

From Table 3, it can be seen that most (106) of the patients were discharged early and particularly in less than 11 days.

**Fig. 3:**
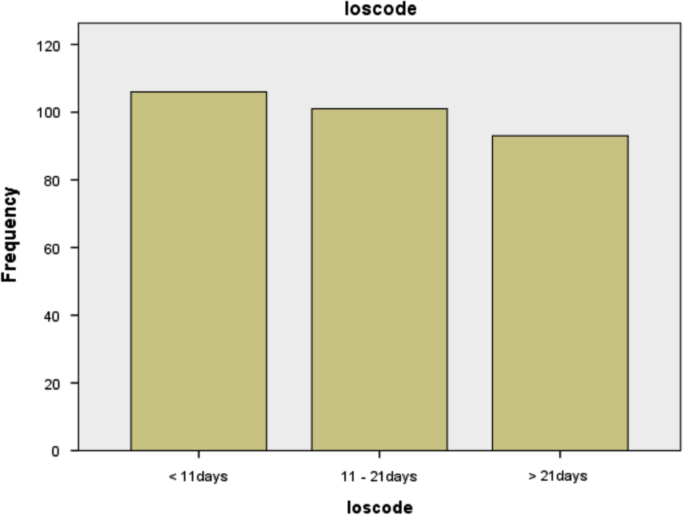
Bar chart showing the classification of length of stay.

The diagrammatic representation is as shown in Fig. 3.

### 1.3 Analysis on the gender of the patients

The information on the gender of the patients is as shown in Table 4.

**Table 4:** Distribution of gender of the patients.

Gender/sex		Frequency	Percent	Cumulative Percent
	Female	275	91.7	91.7
	Male	25	8.3	100.0
	Total	300	100.0	

It can be seen in Table 4 that majority (275) of the patients are females. Also, the table revealed the incidence of breast cancer among male patients.

**Fig. 4:**
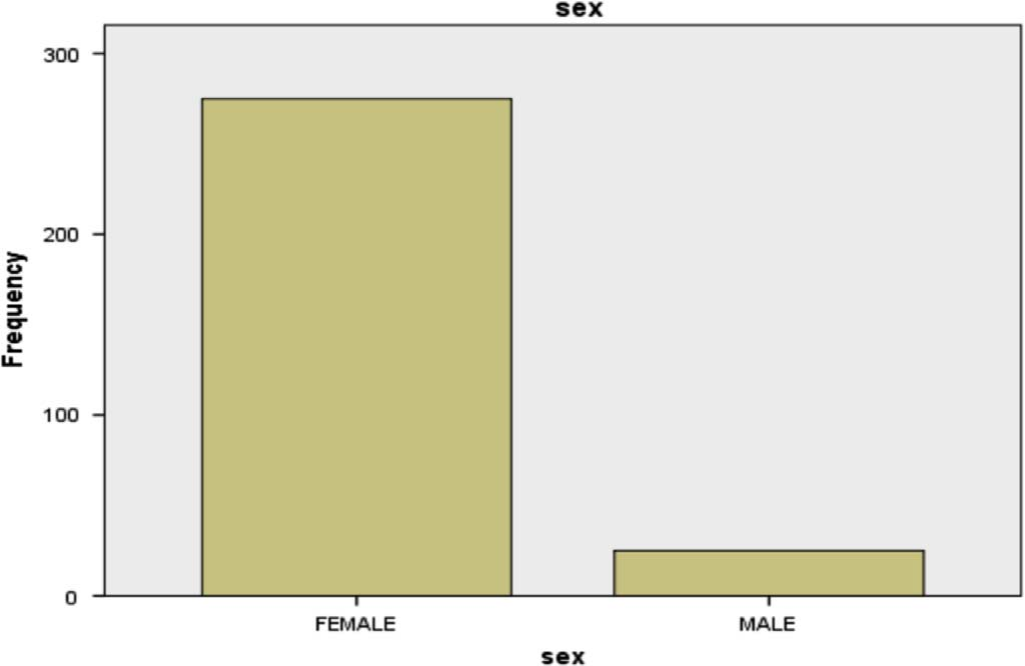
Bar chart showing the distribution of gender.

The information in Table 4 is represented diagrammatically in Fig. 4.

## 2 Experimental design, materials and methods

Research on breast cancer and other form of cancer are intense because of the high fatality rate of the disease if not properly managed. Several aspects of breast cancer has been studied, some of which have generated data sets. The analysis on those data sets is based on the various experimental designs, research materials and referred scientific methods. Some of such areas are: CT images, growth factor levels in incident breast cancer, hormone receptor status, cytokine circulation, secretagogue users in breast cancer treatments, chemokine levels, breast cancer and diabetes mellitus co-infection and treatment, breast cancer and HIV treatment, breast cancer and pregnancy. Others are: proteome analysis, risk factors analysis, breast examination, screening, management and breast cancer awareness, epidemiology, risk assessment tools, treatment options: radiotherapy treatment versus chemotherapy, survival analysis, breast cancer subtypes, biomarkers, socio-cultural barriers to treatment, socio-demographic factors and alternative medicine approach, genetic risk, dietary patterns, early diagnostics and treatment and others [1], [2], [3], [4], [5], [6], [7], [8], [9], [10], [11], [12], [13], [14], [15], [16], [17], [18], [19], [20], [21], [22], [23], [24], [25], [26].

Chi-square test of independence can be used to analyze the data collected, for instance, a cross-tabulation of gender and outcome of the patients at the point of discharge can be classified into a r x c contigency table as shown in Table 5. In this research however, logistic regression analysis was used to analyze the data set. See similar analysis in [27], [28], [29], [30]

**Table 5:** Crosstabulation for gender and outcome of patients.

sex * Outcome Crosstabulation				
Count				
		Outcome		Total
		Alive	Dead	
Sex	female	188	87	275
	male	15	10	25
Total		203	97	300

Table 6 represents the coding for variables length of stay, age, location of cancer, mode of diagnosis and gender of the patients.

**Table 6:** Categorical variable coding.

		Frequency	Parameter coding	
			(1)	(2)
Loscode	< 11days	106	1.00	0.00
	11–21days	101	0.00	1.00
	> 21days	93	0.00	0.00
Agecode	<41years	88	1.00	0.00
	41–55years	115	0.00	1.00
	> 55years	97	0.00	0.00
Location of Cancer	Both breasts	25	1.00	0.00
	Left breast	140	0.00	1.00
	Right breast	135	0.00	0.00
Mode of Diagnosis	Cytological	166	1.00	
	Histological	134	0.00	
sex	Female	275	1.00	
	Male	25	0.00	

Table 7 shows the classification table at step 0.

**Table 7:** Classification Table.

**Classification Table**^**a,b**^					
	Observed		Predicted		
			Outcome		Percentage Correct
			Alive	Dead	

Step 0	Outcome	Alive	203	0	100.0
		Dead	97	0	.0
	Overall Percentage				67.7

Table 8 shows the variables in the equation at Step 0.

**Table 8:** Variables in the equation.

		B	S.E.	Wald	df	Sig.	Exp(B)
Step 0	Constant	−.738	.123	35.797	1	.000	.478

**Block 1: Method = Backward Stepwise (Conditional)**.

Table 9 shows the omnibus tests of model coefficients.

**Table 9:** Tests of model coefficients.

**Omnibus Tests of Model Coefficients**				
		Chi-square	df	Sig.
Step 1	Step	20.742	8	.008
	Block	20.742	8	.008
	Model	20.742	8	.008
Step 2^a^	Step	−.892	2	.640
	Block	19.850	6	.003
	Model	19.850	6	.003
Step 3^a^	Step	−.235	1	.628
	Block	19.616	5	.001
	Model	19.616	5	.001
Step 4^a^	Step	−.461	1	.497
	Block	19.155	4	.001
	Model	19.155	4	.001

a A negative Chi-squares value indicates that the Chi-squares value has decreased from the previous step.

Table 10 shows the model summary using the log-likelihood, Cox & Snell R square and Negelkerke R square.

**Table 10:** Model summary.

**Model Summary**			
Step	-2 Log likelihood	Cox & Snell R Square	Nagelkerke R Square
1	356.872^a^	.067	.093
2	357.764^a^	.064	.089
3	357.998^a^	.063	.088
4	358.459^a^	.062	.086

a Estimation terminated at iteration number 4 because parameter estimates changed by less than .001.

Table 11 shows the variables in the equation from Step 1 to Step 4:

**Table 11:** Variables in the equation.

		B	S.E.	Wald	df	Sig.	Exp(B)	95% C.I.for EXP(B)	
								Lower	Upper
Step 1^a^	sex(1)	−.232	.454	.261	1	.609	.793	.325	1.932
	agecode			9.641	2	.008			
	agecode(1)	−.827	.332	6.194	1	.013	.437	.228	.839
	agecode(2)	−.875	.309	7.996	1	.005	.417	.227	.765
	Location of Cancer			9.209	2	.010			
	Location of Cancer(1)	1.092	.470	5.407	1	.020	2.981	1.187	7.485
	Location of Cancer(2)	.721	.276	6.847	1	.009	2.057	1.198	3.531
	Mode of Diagnosis(1)	−.156	.263	.353	1	.552	.855	.511	1.432
	loscode			.883	2	.643			
	loscode(1)	−.238	.319	.559	1	.455	.788	.422	1.471
	loscode(2)	.031	.316	.010	1	.921	1.032	.555	1.918
	Constant	−.271	.503	.289	1	.591	.763		
									
Step 2^a^	sex(1)	−.220	.453	.237	1	.626	.802	.330	1.948
	agecode			9.669	2	.008			
	agecode(1)	−.827	.331	6.253	1	.012	.437	.229	.836
	agecode(2)	−.871	.309	7.964	1	.005	.419	.229	.766
	Location of Cancer			9.573	2	.008			
	Location of Cancer(1)	1.093	.468	5.460	1	.019	2.983	1.193	7.462
	Location of Cancer(2)	.742	.274	7.323	1	.007	2.100	1.227	3.593
	Mode of Diagnosis(1)	−.166	.263	.397	1	.529	.847	.506	1.418
	Constant	−.359	.459	.613	1	.434	.698		
									
Step 3^a^	agecode			10.684	2	.005			
	agecode(1)	−.852	.326	6.814	1	.009	.427	.225	.809
	agecode(2)	−.898	.304	8.743	1	.003	.407	.225	.739
	Location of Cancer			9.389	2	.009			
	Location of Cancer(1)	1.076	.466	5.325	1	.021	2.933	1.176	7.318
	Location of Cancer(2)	.728	.272	7.154	1	.007	2.072	1.215	3.533
	Mode of Diagnosis(1)	−.178	.261	.461	1	.497	.837	.502	1.398
	Constant	−.528	.303	3.033	1	.082	.590		
									
Step 4^a^	agecode			10.359	2	.006			
	agecode(1)	−.832	.324	6.581	1	.010	.435	.230	.822
	agecode(2)	−.877	.302	8.446	1	.004	.416	.230	.752
	Location of Cancer			9.581	2	.008			
	Location of Cancer(1)	1.114	.463	5.784	1	.016	3.047	1.229	7.554
	Location of Cancer(2)	.722	.272	7.055	1	.008	2.059	1.208	3.509
	Constant	−.640	.256	6.256	1	.012	.528		

a Variable(s) entered on step 1: sex, agecode, LocationofCancer, ModeofDiagnosis, loscode.

Table 12 shows the Hosmer and Lemeshow Test.

**Table 12:** Hosmer and Lemeshow Test.

**Hosmer and Lemeshow Test**			
Step	Chi-square	df	Sig.
1	8.566	8	.380
2	1.502	8	.993
3	1.380	8	.995
4	1.193	5	.946

Table 13 shows the classification table for all the steps; steps 1–4.

**Table 13:** Classification Table.

	Observed		Predicted		
			Outcome		Percentage Correct
			Alive	Dead	
Step 1	Outcome	Alive	187	16	92.1
		Dead	74	23	23.7
	Overall Percentage				70.0
Step 2	Outcome	Alive	193	10	95.1
		Dead	81	16	16.5
	Overall Percentage				69.7
Step 3	Outcome	Alive	180	23	88.7
		Dead	68	29	29.9
	Overall Percentage				69.7
Step 4	Outcome	Alive	180	23	88.7
		Dead	68	29	29.9
	Overall Percentage				69.7

a. The cut value is .500

**Fig. 5:**
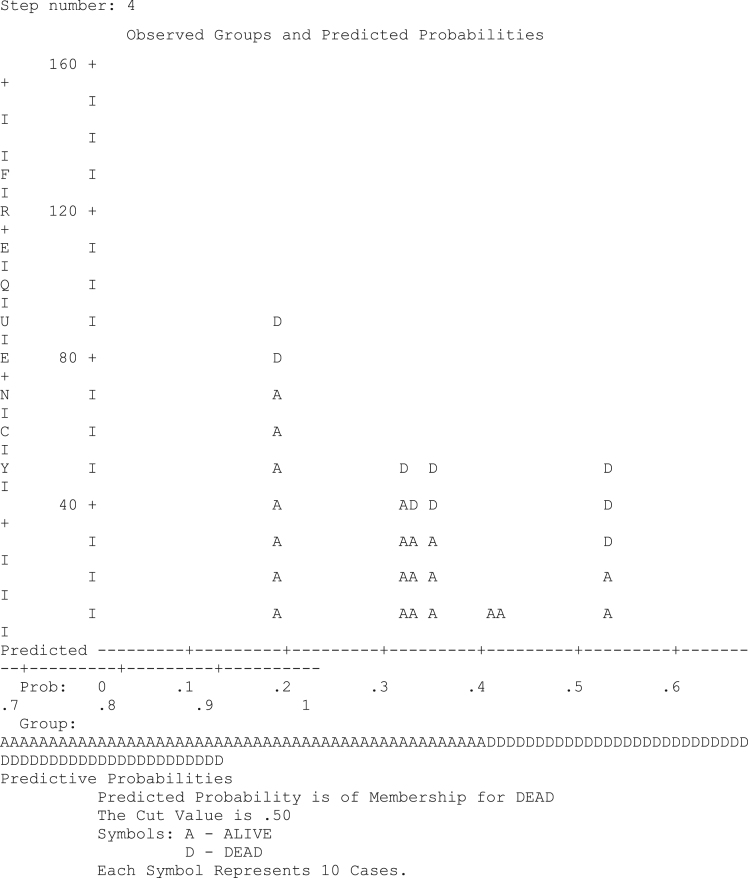
Diagram of predictive probabilities.

The predictive probability is as shown in Fig. 5.

Breast cancer is one of the dangerous diseases. It occurs in both males and females but the incidence is more in females. Based on this present study, the age of the patient and the location of the breast cancer (right breast or left breast) both contribute significantly to whether a patient would survive the breast cancer disease or not.
