# Supplementary material for: Breast cancer patients in Nigeria: Data exploration approach
Source: Data Brief. 2017 Sep 1;15:47–57. doi: 10.1016/j.dib.2017.08.038 (PMC5612794; doi:10.1016/j.dib.2017.08.038)
Supplement: Supplementary file 1 — Supplementary material [file mmc1.pdf]

# COVENANT UNIVERSITY

## COLLEGE OF SCIENCE AND TECHNOLOGY

### DEPARTMENT OF MATHEMATICS

CANAANLAND, KM 10, IDIROKO ROAD
P.M.B 1023, OTA, OGUN STATE, NIGERIA
www.covenantuniversity.edu.ng, mat.covenantuniversity.edu.ng

EXTERNAL MEMO

**To:** Editor, Data in Brief
**From:** Corresponding Author
**Date:** 8th August, 2017
**Subject:** **Conflict of Interest**

I thereby declare the absence of any conflict of interest among the authors.

The authors have read the final draft and unanimously agreed that the paper be sent for review.

Pelumi E. Oguntunde (Ph.D)
Department of Mathematics, Covenant University, Nigeria
Google Scholar: Pelumi E. Oguntunde (Ph.D)
Research Gate: Pelumi Oguntunde
ORCID ID: 0000-0003-3234-4142
SCOPUS ID: 56191957200
